# The Story of the Insane from Year to Year

**Published:** 1899-03-18

**Authors:** 


### The Institutional Workshop.

### THE STORY OF THE INSANE FROM YEAR TO YEAR.

(Eleventh Series.)

### BARNWOOD HOUSE HOSPITAL, NEAR GLOUCESTER.

During the year 1897 the numbers rose from 149 to 155. There were 34 admissions, 7 readmissions, 17 recoveries, and 9 deaths. The recoveries stood at 41·4 per cent. on the admissions, and the deaths at 5·9 per cent. on the average number resident. Of the year's admissions 14 of the patients were supposed to owe their insanity to such moral causes as domestic trouble and mental anxiety, and 22 to hereditary tendency; intemperance in drink not being represented at all. Only 3 of the deaths were due to cerebral diseases, and 2 to consumption. We are pleased to note that "the work of the hospital was carried on peacefully and steadily without the interruption of untoward events," and we may add that the reputation of the hospital is very high. Thirteen patients were admitted at reduced rates, and 11 of these paid sums varying from 10s. to 25s. a week, while 2 were admitted free of charge. These refer to the year's admissions; and we learn further that 4 were maintained gratuitously during the year, 34 paid under 25s., 58 paid from 25s. to 42s., and 26 from 42s. to 63s., while 33 paid 63s. and upwards. We do not learn how many paid sums much under 25s. a week, but it could not exceed 34 out of a total of 150. Making due allowance for the four cases maintained gratuitously, it is not easy to see where the "much charitable work" comes in. Some public asylums admit patients at 25s. a week and others at lower sums, and there is no mention of charity in the case. The Isle of Wight Asylum, for example, is advertising at present for private patients to be admitted into their private patients' block at 25s.; and, unless our memory deceives us, the Dorset County Asylum does the same. The committee record their appreciation of Dr. Soutar's services, and they add a word of praise to the medical assistants, Drs. Townsend and Kerr.

### CHESHIRE COUNTY ASYLUM AT MACCLESFIELD.

On January 1st, 1897, this asylum contained 678 patients, and on the last day of the year the numbers had risen to 705. There were 137 first admissions, 25 readmissions, 51 recoveries, and 61 deaths. These numbers show that the recoveries, calculated in the usual way, stand at 31·6 per cent. on the admissions, and the deaths at 9 per cent. on the average number resident. These percentages are almost the same as the average since the opening of the asylum in 1871. Twenty of the deaths were caused by cerebral and spinal diseases, 6 by inflammation of the lungs, and 10 by consumption. Of the admissions, 26 owed their insanity to various moral causes, 33 to intemperance in drink, and 25 to hereditary influence. Ten were congenital cases, and in 35 previous attacks were the assigned causes. It would seem that the asylum is practically full; but we do not notice any reference to additions in the visitors' report—indeed, they seem to have handed money over to the county which must speedily be wanted for building purposes. It cannot be gainsaid that asylum committees have, in the main, admirably discharged their duties; but in the question of providing for the future wants of the county, they are almost invariably late, and thus place quite unnecessary burdens on the ratepayers. We are glad to note that the assistant medical officers (Drs. Laing and Scanlon) continue their instructions to the attendants and nurses, and that large numbers of the staff have passed examinations in first aid and sick nursing. In the next annual report we hope to learn that Dr. Sheldon has inaugurated a "three years' system of training" on the basis of the hospital plan, and that by the end of three years a large proportion of his staff will be in possession of "the Macclesfield certificate," and, from our knowledge of Dr. Sheldon, we believe such a certificate would be of real value. We congratulate William Weston, the painter attendant, on having obtained a pension of £26 a year. This is not very much to live on, and we hope Mr. Weston was thoughtful enough to join the Royal National Pension Fund for Nurses. We make no apology for quoting the following words from Dr. Sheldon's report: "There can be no doubt," he says, "that the prospect of even a small pension is a strong incentive to remain in a service which of all public services is the most painful and disagreeable." We have also to congratulate the storekeeper on having obtained a pension of £100 a year, which his length of service fully merits. In connection with the resignation involved in this we cannot say we think the visitors have acted wisely in joining the duties of the clerk of the asylum and storekeeper. These duties are so different that it must be extremely difficult to find a man who understands both and who will perform both. The committee record "their satisfaction with the manner in which the asylum is managed by Dr. Sheldon." The cost per head per week is 8s. 9⅞d.

### FIFE AND KINROSS DISTRICT ASYLUM.

Here the numbers rose from 484 to 516. There were 99 first admissions, 32 readmissions, 44 recoveries, 28 discharged relieved, and 27 deaths. The recoveries stand at 35·11 per cent. on the admissions, and the deaths at 9·59 per cent. on the average number resident. Of the year's deaths 14 were caused by cerebral and spinal diseases and 5 by consumption. Domestic trouble, adverse circumstances, &c., account for 22 of the year's admissions, intemperance in drink for 18, and hereditary influence for 40. There has been a steady increase in the number of admissions. During the decade ending 1890 the average annual admission-rate was 87, during the five years ending 1895 it was 101, and since then it has risen to 128. The visitors think some of this increase is due to the transference of senile cases to the asylum. Dr. Turnbull does not say whether he thinks there is an increase of insanity amongst us nowadays, but he tells us, what we have known for a long time, that there is an increased demand for asylum accommodation. The additions built at Fife and Kinross are already full, and once more unwelcome additions must make their appearance. Wise is that committee who anticipate the wants of their district, for they can always clear large sums from the shortcomings of their neighbours. The visitors say that "care and attention are unremittingly given" by Dr. Turnbull. The average cost per head per annum is £24 11s.

### LEICESTER AND RUTLAND COUNTY ASYLUM.

Curiously enough there was a decrease in the number of patients in this asylum during 1897, there having been 505 on January 1st, and only 481 on the last day of December; but this decrease was owing to the transference of 35 patients to other asylums, and it would seem that even yet there are several cases too many in the asylum. There were 86 first admissions, 15 readmissions, 33 recoveries, and 43 deaths. The recoveries stand at 33 per cent. on the admissions, and the deaths at 8·6 on the average number resident. Both of these percentages are below the averages in county asylums. Of the deaths 17 were caused by cerebral and spinal diseases, 1 by pneumonia, and 11 by consumption.

Of the year's admissions 18 owed their insanity to domestic trouble, anxiety, and other moral causes, 10 to intemperance in drink, 26 were hereditary, and 10 were congenital. Some inconvenience was caused by the crowded state of the asylum. As already stated 35 patients have been boarded out at other asylums, and 20 beds had to be taken from the male side and given to the women. It has been decided to remove the asylum to another site. The weekly charge to the unions is 8s. 9d. per head.

